# *Ab Initio* Protein Structure Prediction Using Pathway Models

**DOI:** 10.1002/cfg.305

**Published:** 2003-07

**Authors:** Xin Yuan, Yu Shao, Christopher Bystroff

**Affiliations:** Department of Biology Rensselaer Polytechnic Institute Troy NY 12180 USA

## Abstract

*Ab initio* prediction is the challenging attempt to predict protein structures based only on sequence information and without using templates. It is often divided into two distinct sub-problems: (a) the scoring function that can distinguish native, or native-like structures, from non-native ones; and (b) the method of searching the conformational space. Currently, there is no reliable scoring function that can always drive a search to the native fold, and there is no general search method that can guarantee a significant sampling of near-natives. Pathway models combine the scoring function and the search. In this short review, we explore some of the ways pathway models are used in folding, in published works since 2001, and present a new pathway model, HMMSTR-CM, that uses a fragment library and a set of nucleation/propagation-based rules. The new method was used for *ab initio* predictions as part of CASP5. This work was presented at the Winter School in Bioinformatics, Bologna, Italy, 10–14 February 2003.


**Comparative and Functional Genomics**
*Comp Funct Genom* 2003; **4**: 397–401.
Published online 21 July 2003 in Wiley InterScience (www.interscience.wiley.com). **DOI:** 10.1002/cfg.305

**Conference Review**

### *Ab initio* protein structure prediction using pathway models

Xin Yuan, Yu Shao and Christopher Bystroff*
*Department of Biology, Rensselaer Polytechnic Institute, Troy, NY 12180, USA*

*Correspondence to: Christopher Bystroff, Department of Biology, Rensselaer Polytechnic Institute, Troy, NY 12180, USA. E-mail: bystrc@rpi.edu*

Received: 22 May 2003
Revised: 30 May 2003
Accepted: 2 June 2003

### Abstract

***Ab initio*** **prediction is the challenging attempt to predict protein structures based only on sequence information and without using templates. It is often divided into two distinct sub-problems: (a) the scoring function that can distinguish native, or native-like structures, from non-native ones; and (b) the method of searching the conformational space. Currently, there is no reliable scoring function that can always drive a search to the native fold, and there is no general search method that can guarantee a significant sampling of near-natives. Pathway models combine the scoring function and the search. In this short review, we explore some of the ways pathway models are used in folding, in published works since 2001, and present a new pathway model, HMMSTR-CM, that uses a fragment library and a set of nucleation/propagation-based rules. The new method was used for *ab initio* predictions as part of CASP5. This work was presented at the Winter School in Bioinformatics, Bologna, Italy, 10–14 February 2003. Copyright © 2003 John Wiley & Sons, Ltd.**

### Introduction

Protein structure prediction methods have implicit underlying principles that fall into two categories: evolution and folding. Evolution-based methods seek to find conserved sequence patterns, while folding methods simulate the physical process of folding. A folding pathway is a time series of protein folding events. Most molecular simulation methods, including molecular dynamics (MD) and Monte Carlo (MC), create a pathway implicitly. Other methods enforce certain characteristics of the folding events during the simulation, including some genetic algorithms, neural nets, and a new rule-based approach.

### Detailed molecular representations

The MD approach to folding draws its strength from the fundamental nature of its physics-based energy function. Unfortunately, unless simplified models can be used, long simulations are still far too costly to be practical. Head-Gordon and Crivelli have developed the global optimization methods called 'Stochastic Perturbation with Soft Constraints'. The atom-based energy function and novel hydrophobic solvation function of their MD approach is able to discriminate against misfolds. However, the method is still computationally expensive, and it needs improvement in $\beta$-strand and loop matching [5].

In Beveridge's protocol, they combined an AMBER united atom empirical energy functions, a GBSA (generalized born/solvent accessibility) for solvent dielectric polarization, Van der Waals and cavitation effects, and a multiple-copy MCSA (Monte Carlo simulated annealing) searching scheme, which is able to escape to some extent from meta-stable local minimum. The results show that the method is able to recover the structures of test cases within 6.0 Å RMS [12].

### Simplified models and lattice simulations

In Gibbs's *ab initio* method, the protein conformation is represented using backbone torsion

Copyright © 2003 John Wiley & Sons, Ltd.

angles and fixed side chains. An evolutionary Monte Carlo algorithm is developed to search through this restricted conformational space. The simple physiochemical force field based on hydrophilic, hydrophobic, steric and hydrogen-bonding potentials is used to assess the energies. The 3D structures of polypeptide chains up to 38 residues have been accurately predicted [7].

Scheraga's group used the hierarchical approach for global optimization of an off-lattice simplified chain, with a modified united-residue (UNRES) force field and their conformational space annealing (CSA) global optimization procedure. Good results have been obtained for both a four- and a three-helix protein [17].

LINUS, developed by Rose's group, is a Monte Carlo program that emphasizes the role of steric interactions and conformational entropy. Simple scoring functions represent the hydrogen bonds and hydrophobic interactions [19].

Lattice-based studies represent proteins on a cubic or tetrahedral lattice, and this reduces the conformational space enormously, making even exhaustive simulations possible for short chains (e.g. 27 residues).

The recent face-centred cubic lattice model in Skolnick's group includes the interactions between hydrophobic residues, repulsive interactions between hydrophobic and polar residues, and orientation-dependent polar–polar interactions. Their replica exchange Monte Carlo method is able to reproduce a cooperative all-or-none folding transition and the cooperative formation of secondary structure upon the folding transition [14].

Skolnick also proposed a lattice-based parallel hyperbolic sampling (PHS) Monte Carlo algorithm through the logarithmic flattening of the local high-energy barriers by an inverse hyperbolic sine function, which can overcome the local minima trapping and speed up the 'thermalization' of the protein folding process (meaning the time spent to reach equilibrium). They applied the method to the side chain-only protein model and were able to identify much lower energy structures and explore a larger conformational space than the replica-sampling MC method. They also pointed out that the minimum relative RMSD (mrRMSD) is more favourable than lowest-energy for prediction quality. The drawback of PHS is that for a relatively smooth energy landscape system it might be less efficient than other methods [24].

### Fragment libraries

The hierarchical condensation of a polypeptide may be roughly modelled by simulations that draw from a fragment library. Each fragment is a preferred conformer for a segment of the chain, usually defined by sequence statistics or motif patterns. Fragment library simulations leapfrog the earliest steps in folding, that being the formation of local structure.

Levitt's group constructed proteins from different-sized fragment libraries (four to seven residues) using a simulated-annealing $k$-means clustering method. Their discrete approximation model is able to achieve 1 Å accuracy with lower complexity for four- and five-residue fragments. However, the complexity for longer fragments still needs to be improved [11]. Their study demonstrates that it is sufficient to use fragments for protein structure simulations. This has relevance to the work of several other groups, including Karplus [10], Baker [1], Jones [9] and Bystroff [18], all of whom use the fragment libraries for simulations.

In general, fragment library simulations use knowledge-based potentials and a simplified side chain representation while swapping fragments drawn from a library. The first such program was Baker's ROSETTA algorithm [1], automated in Bystroff's I-sites/Rosetta server [3], which explores conformation space using MC simulated annealing and a Bayesian knowledge-based potential. Jones's FRAGFOLD starts with a library of common supersecondary units and also applies simulated annealing [9]. Karplus's Undertaker uses HMMs (and other sources) to build a fragment library and optimizes the 'cost of burial' [10]. Fragment library simulations have had perhaps the broadest success in *ab initio* prediction in the last three CASP meetings.

### HMMSTR-CM: rule-based folding in 2D

HMMSTR-CM is a new algorithm based on HMMSTR [4] that was used for predicting contact maps in CASP5. The approach is not a simulation but a set of knowledge-based potentials and rules for building a protein contact maps. Nucleation/condensation-type folding pathways are encoded in the rule set. A contact map is a low-resolution, 2D representation of a protein's 3D

Copyright © 2003 John Wiley & Sons, Ltd. 

structure. Contact maps have been used as a tool for protein structure prediction [6,13,15], particularly because the representations of 3D structure that they produce are data that can easily be mined [8,22,23]. Contact maps may be projected into 3D using existing algorithms [2,20,21].

For a given protein sequence, its contact potential map (Figure 1a) is calculated using HMMSTR, a hidden Markov model for local sequence structure correlations. A contact potential is the negative log-likelihood of a contact between a pair of Markov states, one at each of two positions in the sequence. The Markov states for each position in the sequence are assigned using the forward/backward algorithm [16]. In Figure 1a, a low contact potential is coloured red and a high contact potentials are blue. Secondary structures, which can be predicted directly by HMMSTR, can also be identified in the contact potential map, e.g. strong $i$ to $i+4$ contacts indicate predicted helix, and predicted $\beta$-strands tend to have low contact potentials with other strands. In Figure 1a, three helices and four (or five) $\beta$-strands can be identified.

HMMSTR-CM initially overpredicts contacts, with few false negatives. Thus, the accuracy of the *ab initio* approach depends on the accuracy of pruning false positives. A nucleation propagation folding pathway scheme is used to find the true contacts. Its success depends strongly on the choice of the initial nucleation site. The strategy of the

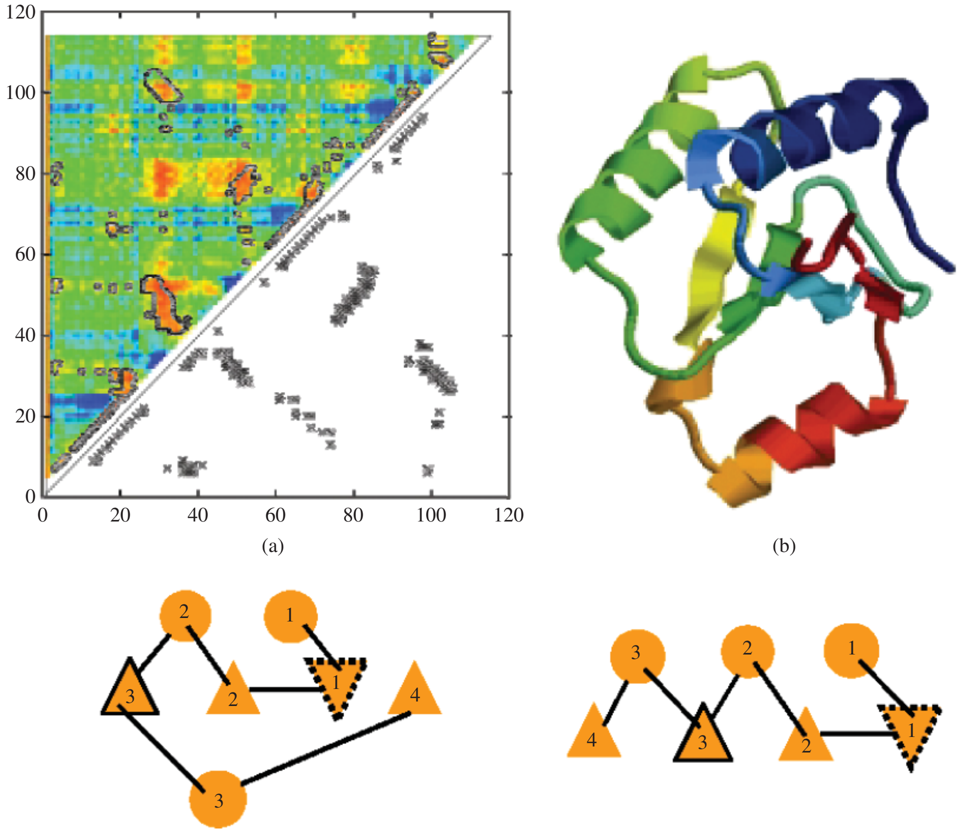

**Figure 1.** (a) The upper left triangle is the superposition of the predicted contact maps of T0130 on top of its contact potential map. The predicted contacts are represented by the black outlines. The color of the contact potential map ranges from red (low energy) to blue (high). The lower right triangle is the contact map of the true structure of T0130. (b) Rasmol image of the true 3-D structure of T0130. (c) The correct TOPS diagram. The circles represent helices and triangles represent strands. The dotted line indicates the non-polar strand and the solid line indicates the amphipethic strand. (d) The wrong TOPS diagram. Reproduced from Shao Y, Bystroff C (2003), 'Predicting inter-residue contacts using templates and pathways' in *Proteins, Structure, Function and Genetics* by permission of Wiley-Liss, Inc., a subsidiary of John Wiley & Sons, Ltd

Copyright © 2003 John Wiley & Sons, Ltd. 

**Table 1.** Rules for folding in contact map space. Contacts are assigned if they have the lowest contact free energy and satisfy the following rules

| Physicality and propagation rules |
|---|
| 1. *Propagation rule*: residues $i$ and $j$ are separated by no more than five sequence positions or are both within five residues of the same block of previously defined contacts |
| 2. *Maximum neighbour rule*: one residue can have at the most 12 contacts |
| 3. *Maximum mutual contact rule*: if residue $i$ and $j$ are in contact, there are at the most six residues in contact with both $i$ and $j$ |
| 4. *β-pairing rule*: a β-strand can be in contact with at the most two other β-strands |
| 5. *β-sheet rule*: any two pairing strands are either parallel or antiparallel |
| 6. *Helix mutual contact rule*: a residue cannot be in contact at the same time with the residues on the opposite sides of a helix |
| 7. *Helix rule*: within a helix, only the contact between residue $i$ and $i + 4$ is allowed |
| 8. *β-rule*: no contact is allowed within any strand |
| 9. *Right-hand crossover rule*: crossovers between parallel strands of the same sheet (paired or not) are right-handed (especially if the crossover contains a helix) |
| 10. *Helix crowding rule*: if a helix can go to either side of a sheet, it picks the side with fewer crossovers |
| 11. *Strand burial rule*: if a strand can pair with either of two other strands, it chooses the one that is more non-polar |

prediction is: (a) predict the secondary structure; (b) choose a folding nucleation site by assigning local contacts; (c) propagate from the nucleation site by assigning or removing contacts, based on physicality and propagation rules (Table 1). The prediction is finished when all pairs are assigned either a contact or non-contact, and when none of the rules are violated.

### Results from CASP5

Here we will discuss one example of HMMSTR-CM prediction of a CASP5 target. Summaries of prediction methods, including this one, can be found in a special edition of the journal *Proteins, Structure, Function and Genetics* this year [18], to be dedicated to the CASP5 prediction experiment.

Target T0130 has 116 residues arranged in a three-layer $\alpha/\beta$ sandwich. The contact potential map is shown in Figure 1a. By choosing different nucleation sites, we found more than one way to derive a physically possible topology. In this case, we selected to start the pathway with $\beta_2\alpha_2\beta_3$. The following is the sequence of operations that built the prediction. This sequence of events is the predicted folding pathway:

1. Parallel $\beta$ contacts were assigned between $\beta_2$ and $\beta_3$.
2. Anti-parallel contacts were assigned to $\beta_1$ and $\beta_2$. All other $\beta$ contacts to $\beta_2$ were pruned.
3. There were two ways to make a right-handed crossover from $\beta_3$ to $\beta_4$ (Figure 1c–d). Since $\beta_1$ is more hydrophobic than $\beta_3$, we paired $\beta_1$ with $\beta_4$. All other $\beta$ contacts to $\beta_1$ were pruned, and contacts between $\alpha_2$ and $\alpha_3$ were pruned since they are now on opposite sides of the sheet.
4. $\alpha_1$ must be on the opposite side of the sheet from $\alpha_{3,}$ since $\alpha_3$ extends across the sheet. Contacts were assigned between $\alpha_1$ and $\alpha_2$.

The completed TOPS diagram and contact map accurately match the true structure (Figure 1b). The prediction has 42% contact coverage and 29% accuracy. However, if we count near-misses (±one residue), the coverage is 75% and the accuracy is 57%. Note that the long-range contacts between the $\beta_1$ and $\beta_4$ were correctly predicted. Long-range contacts are difficult to predict using purely statistical methods.

Identification of the folding nucleation site is the critical step in this approach. Once the nucleation site is chosen, the subsequent contact assignments are often unambiguous. The choice of the nucleation site in T0130 was relatively easy. Only one of the three parallel $\beta\alpha\beta$ units had a high score. The hairpin between $\beta_1$ and $\beta_2$ would also be a correct choice, but the selection of $\beta_2\alpha_2\beta_3$ eliminated more of the potential incorrect folding pathways. This prediction turned out to be topologically correct. In other cases, the wrong structure was chosen for the nucleation site, and the algorithm failed. When the correct nucleation site was assigned retrospectively, the correct topology could be identified, but this has not yet been cross-validated.

### Summary

Simulating the physical process of protein folding has taken many algorithmic forms, distinguished by the differing levels of detail in the representation of the model. Detailed all-atom representations continue to be popular, while simplified models have proved to be successful in blind predictions.

Copyright © 2003 John Wiley & Sons, Ltd.

Pathways have recently been defined for a 2D contact map representation, and this approach shows potential for modelling the folding process without simulations.

### References

1. Bonneau R, Strauss CE, Rohl CA, *et al*. 2002. *De novo* prediction of three-dimensional structures for major protein families. *J Mol Biol* **322**: 65–78.
2. Brunger AT, Clore GM, Gronenborn AM, Karplus M. 1986. Three-dimensional structure of proteins determined by molecular dynamics with interproton distance restraints: application to crambin. *Proc Natl Acad Sci USA* **83**: 3801–3805.
3. Bystroff C, Shao Y. 2002. Fully automated *ab initio* protein structure prediction using I-SITES, HMMSTR and ROSETTA. *Bioinformatics* **18**(suppl 1): S54–S61.
4. Bystroff C, Thorsson V, Baker D. 2000. HMMSTR: A hidden Markov model for local sequence–structure correlations in proteins. *J Mol Biol* **301**: 173–190.
5. Crivelli S, Eskow E, Bader B, *et al*. 2002. A physical approach to protein structure prediction. *Biophys J* **82**: 36–49.
6. Fariselli P, Olmea O, Valencia A, Casadio R. 2001. Progress in predicting inter-residue contacts of proteins with neural networks and correlated mutations. *Proteins* **45**(S5): 157–162.
7. Gibbs N, Clarke AR, Sessions RB. 2001. *Ab initio* protein structure prediction using physicochemical potentials and a simplified off-lattice model. *Proteins* **43**: 186–202.
8. Hu J, Shen X, Shao Y, Bystroff C, Zaki MJ. 2002. Mining Protein Contact Maps, 2nd BIOKDD workshop on data mining in bioinformatics 2002, Edmonton, Canada. **[http://www.cs.rpi.edu/~zaki/PS/BIOKDD02.pdf]**
9. Jones DT. 2001. Predicting novel protein folds by using FRAGFOLD. *Proteins* **45**(S5): 127–132.
10. Karplus K, Karchin R, Hughey R. 2003. Unifying secondary structure, fold-recognition, and new fold methods for protein structure prediction. **http://www.cse.ucsc.edu/~karplus/papers/sam-undertaker.pdf** (accessed 12 May 2003).
11. Kolodny R, Koehl P, Guibas L, Levitt M. 2002. Small libraries of protein fragments model native protein structures accurately. *J Mol Biol* **323**: 297.
12. Liu Y, Beveridge DL. 2002. Exploratory studies of *ab initio* protein structure prediction: multiple copy simulated annealing, AMBER energy functions, and a generalized born/solvent accessibility solvation model. *Proteins* **46**: 128–146.
13. Olmea O, Rost B, Valencia A. 1999. Effective use of sequence correlation and conservation in fold recognition. *J Mol Biol* **293**: 1221–1239.
14. Pokarowski P, Kolinski A, Skolnick J. 2003. A minimal physically realistic protein-like lattice model: designing an energy landscape that ensures all-or-none folding to a unique native state. *Biophys J* **84**: 1518–1526.
15. Pollastri G, Baldi P. 2002. Prediction of contact maps by GIOHMMs and recurrent neural networks using lateral propagation from all four cardinal corners. *Bioinformatics* **18**(suppl 1): S62–S70.
16. Rabiner LR. 1989. A tutorial on hidden Markov models and selected applications in speech recognition. *Proc IEEE* **77**: 257–286.
17. Saunders JA, Gibson KD, Scheraga HA. 2002. *Ab initio* folding of multiple-chain proteins. *Pac Symp Biocomput* **7**: 601–612.
18. Shao Y, Bystroff C. 2003. Predicting inter-residue contacts using templates and pathways. *Proteins Struct Funct Genet* (in press).
19. Srinivasan R, Rose GD. 2002. *Ab initio* prediction of protein structure using LINUS. *Proteins* **47**: 489–495.
20. Taylor WR, Aszodi A. 1994. Building protein folds using distance geometry: towards a modeling and prediction method. In *The Protein Folding Problem and Tertiary Structure Prediction*, Merz KM, Le Grand SM (eds). Birkhauser: Boston, MA.
21. Vendruscolo M, Kussell E, Domany E. 1997. Recovery of protein structure from contact maps. *Fold Des* **2**: 295–306.
22. Zaki MJ, Bystroff C. 2001. Mining residue contacts in proteins. In *Data Mining for Scientific and Engineering Applications*, Grossman R, Kamath C, Kegelmeyer P, Kumar V, Namburu R (eds). Kluwer Academic: Boston, MA; 141–164.
23. Zhang C, Kim SH. 2000. Environment-dependent residue contact energies for proteins. *Proc Natl Acad Sci USA* **97**: 2550–2555.
24. Zhang Y, Kihara D, Skolnick J. 2002. Local energy landscape flattening: parallel hyperbolic Monte Carlo sampling of protein folding. *Proteins* **48**: 192–201.

Copyright © 2003 John Wiley & Sons, Ltd. 
